# Comparison of effects of propofol versus sevoflurane for patients undergoing cardiopulmonary bypass cardiac surgery

**DOI:** 10.12669/pjms.35.4.1279

**Published:** 2019

**Authors:** Shaoqun Tang, Wei Huang, Kun Zhang, Wei Chen, Tao Xie

**Affiliations:** 1 Shaoqun Tang, Department of Anesthesiology, Renmin Hospital of Wuhan University, Wuhan 430060, Hubei Province, China; 2 Wei Huang, Department of Neurology, Taihe Hospital Hubei University of Medicine, Shiyan, Hubei 442000, China; 3 Kun Zhang, Department of Anesthesiology, Jingzhou Central Hospital, The Second Clinical Medical College, Yangtze University, Jingzhou, Hubei, 434020, P.R. China; 4 Wei Chen, Department of Anesthesiology, The first people’s hospital of Jingzhou, The first Clinical Medical College, Yangtze University, Jingzhou, Hubei, 434020, P.R. China; 5 Tao Xie, Department of Anesthesiology, Jingzhou Central Hospital, The Second Clinical Medical College, Yangtze University, Jingzhou, Hubei, 434020, P.R. China

**Keywords:** Propofol, Sevoflurane, Cardiopulmonary bypass, Cardiac surgery

## Abstract

**Objective:**

To compare the effects of propofol versus sevoflurane on the outcomes of patients undergoing cardiac surgery with cardiopulmonary bypass (CPB).

**Methods:**

A total of 110 patients undergoing cardiac surgery with CPB in our hospital from January 2015 to June 2017 were randomly divided into 2 groups (n=55): Group A, in which anesthesia was maintained with sevoflurane, and Group B, in which anesthesia was maintained with propofol. The MMSE score before and after operation, perioperative laboratory index, incidence of postoperative cognitive dysfunction (POCD) and incidence of adverse events between the two groups were compared.

**Results:**

The MMSE score was significantly higher in Group B than in Group A after anesthesia (p<0.05). Serum levels of the brain injury markers neuron-specific enolase, S100β and matrix metalloproteinase 9 were significantly lower in Group B than in Group A (p<0.05). POCD incidence at 12 hour and 24 hour after operation was significantly lower in Group B than in Group A (p<0.05). There were no significant differences in the incidence of low cardiac output and thoracotomy bleeding between two groups.

**Conclusion:**

Compared with sevoflurane, the use of propofol during cardiac surgery with CPB can efficiently improve postoperative cognitive function without increasing the risk of adverse reactions.

## INTRODUCTION

Studies have shown that the incidence of postoperative cognitive dysfunction (POCD) in surgical patients is closely related to anesthesia and intraoperative trauma.^1^ In particular, the incidence of POCD in patients undergoing cardiac surgery with cardiopulmonary bypass (CPB) is significantly higher than that of other patients. Globally, scholars have confirmed that these patients have a higher risk of central nervous system injury due to factors such as hypothermia and cerebral venous pressure fluctuations; thus, the level of intelligence is significantly reduced, and the long-term prognosis is seriously affected.^2^

Recent studies have shown that the application of a rational anesthetic program can help alleviate the cerebral blood oxygen supply in patients and protect the central nervous system.^3^ Propofol and sevoflurane are common anesthesia maintenance drugs for patients undergoing cardiac surgery with CPB, but there is no clear conclusion about which one can reduce postoperative cognitive dysfunction. This study aimed to compare the effects of propofol and sevoflurane on the cognitive function and PCOD of patients undergoing cardiac surgery with CPB.

## METHODS

This study was approved by Institute Ethics Committee, and all patients and their family members provided informed consent. A total of 110 patients undergoing cardiac surgery with CPB in our hospital were chosen in the period from January 2015 to June 2017; these patients met the following inclusion criteria: undergoing cardiac surgery; under 75 years old; and ASA grading Grade I-II.^4^ These patients had no history of POCD; their BMI was less than 25 kg/m^2^; they had no mental disease; they had no severe organ dysfunction; and their MMSE score was less than 24. The eligible patients were randomly divided into two groups (n=55).

The study was approved by the Institutional Ethics Committee of our hospitals, and written informed consent was obtained from all participants.

### Operation

The anesthesia induction program was as follows: midazolam 0.05-0.1 mg/kg; sufentanil 0.8-1.5 µg/kg; vecuronium bromide 0.1-0.2 mg/kg; and etomidate 0.1-0.5 mg/kg. A tracheal cannula was used for mechanical ventilation of the operation; oxygen concentration was 30-50%, and the tidal volume was 5-8 ml/kg. The anesthesia maintenance program was as follows: sufentanil 0.6-1.0 µg/(kg·h); and cisatracurium besylate 2 µg/(kg·min). In Group A, sevoflurane inhalation was used to maintain end-expiratory and end-effluent concentrations of 1%-3%. In Group B, a propofol venous pump was used; the concentration in the blood was 0.5-2.0 µg/ml, and the bispectral index of EEG was 40-55 during the operation.

### Outcomes

MMSE scoring was applied to evaluate cognitive function.^4^ A higher score indicated better cognitive function. Serum levels of neuron-specific enolase (NSE), S100β and matrix metalloproteinase 9 (MMP9) were detected by ELISA kits (Quanhui Biotechnology Co., Ltd, Zhuhai, China). The incidence of POCD after operation was recorded using the criteria previously described.^5^

### Statistical Analysis

All data were analyzed by SPSS 18.0 software. The measurement data are expressed as the mean ± standard deviation and analyzed by t-test. The enumeration data were expressed as percentage (%) and analyzed by χ^2^ test. *p*< 0.05 was considered to be a significant difference.

## RESULTS

### Comparison of general data between the two groups

Group A included 29 male patients and 26 female patients, with an average age of 64.10±5.67 years old, average BMI of 24.52±1.97 kg/m^2^, average anesthesia time of 215.46±36.95 minutes, average CPB time of 83.71±11.24 min and average operation time of 190.24±35.68 minutes. In terms of educational background, Group A included 18 patients at the primary school level, 30 patients at the middle school level and 7 patients at the junior college level and above. Group B included 21 male patients and 24 female patients, with an average age of 64.46±5.72 years old, average BMI of 24.59±2.00 kg/m^2^, average anesthesia time of 218.61±37.03 minutes, average CPB time of 83.50±11.19 min and average operation time of 193.02±35.59 minutes. In terms of educational background, Group B included 15 patients at the primary school level, 31 patients at the middle school level and 9 patients at the junior college level and above. Neither groups had significant differences for the above data (*p*>0.05).

The MMSE score of Group B after operation was significantly higher than that of Group A (*p*<0.05, Table-I). Next, we detected serum biomarkers for brain injury, such as NSE, S100β and MMP9. The results showed that serum levels of these biomarkers in Group B at 0 h, 6 h and 12 hour after the operation were significantly lower compared to those of Group A (*p*<0.05, Table-II). The POCD incidence of Group B at 12 hour and 24 hour after operation was significantly lower than that of Group A (*p*<0.05, Table-III).

**Table I:** Comparison of MMSE score before and after operation between two groups.

Group	N	Before operation	24 h after operation
A	55	28.87±4.63	24.30±3.77
B	55	29.15±4.70	28.74±4.53*

* Compared with Group A, p<0.05; ΔCompared with before operation, p<0.05

**Table II:** Comparison of serum levels of biomarkers between two groups.

Group	Point of time	MMP-9	NSE	S100β
A (n=55)	Before operation	51.93±7.61	8.80±0.71	0.25±0.05
	Immediately after operation	77.35±9.25#	21.74±2.84#	0.87±0.11#
	6 h after operation	71.75±9.58#	17.14±2.32#	0.69±0.09#
	12 h after operation	59.74±5.56#	16.32±2.14#	0.35±0.07#
	24 h after operation	52.86±6.34	9.10±0.92	0.20±0.04
B (n=55)	Before operation	51.90±7.52	9.44±0.78	0.28±0.06
	Immediately after operation	72.35±11.17#,*	18.10±2.12#,*	0.72±0.09#,*
	6 h after operation	65.13±8.10#,*	14.59±1.66#,*	0.53±0.07#,*
	12 h after operation	55.86±5.61#	12.16±1.49#,*	0.36±0.06#
	24 h after operation	50.25±6.13	8.86±0.88	0.24±0.04

* Compared with Group A, p<0.05; # Compared with before operation, p<0.05

**Table III:** Comparison of POCD incidence between two groups.

Group	N	12 h after operation	24 h after operation
A	55	12 (21.82%)	14 (25.45%)
B	55	5 (9.09%)*	6 (10.91)*

* Compared with Group A, p<0.05

### Comparison of adverse event incidence between the two groups

There was no significant difference in the incidence of low cardiac output and thoracotomy bleeding between the two groups (*p*>0.05, Table-IV).

**Table IV:** Comparison of adverse event incidence between two groups.

Group	N	Low cardiac output	Thoracotomy bleeding
A	55	0 (0.00%)	2 (3.64%)
B	55	1 (1.82%)	3 (5.45%)

## DISCUSSION

The risk of central nervous system injury is very high after operation for the following reasons: the brain is at hypoperfusion level for a long time during the process of cardiac surgery under CPB; partial microembolus forms; inflammatory response is hyperactive; and the range of temperature variation is large.^6^ The incidence of postoperative neurological complications for such patients can reach 2.3-9.6%.^7^ POCD after cardiac surgery with CPB is considered to be the main manifestation of intraoperative brain damage. The age, renal function, anesthesia scheme, and other underlying diseases are important factors influencing the occurrence of POCD.^8^ In recent years, foreign clinical studies have shown that^9^ a reasonable adjustment of the anesthesia maintenance drug program can effectively reduce the risk of postoperative POCD and reduce damage to central nervous system function. Cell culture experiments confirm that^10^,^11^ volatile anesthetics (isoflurane, sevoflurane, desflurane) can induce apoptosis and increase the formation of beta-amyloid protein. Similarly, with the increase of desflurane dosage, the degree of oligomerization of beta-amyloid protein also increases. Therefore, some scholars propose reducing the dosage of inhalation anesthetics or replacing inhalation anesthesia with intravenous anesthetics to protect patients’ cognitive function.

The serum levels of MMP-9, S100β and NSE have been indicated to reflect cerebral injury and to predict long-term prognosis of patients after the operation.^12^ In this study, we found that the serum levels of these markers were significantly lower in Group B at 0 h, 6 h and 12 h after operation than they were in Group A (*p*<0.05). In addition, postoperative MMSE scores of Group B were significantly higher than those of Group A (*p*<0.05). These results suggest that propofol contributes to relieving cognitive injury in patients undergoing cardiac surgery with CPB and has certain protective effects for the central nervous system. S100β protein is a kind of connective protein secreted by astrocytes and Schwann cells and reflects the status of the blood brain barrier. S100β levels were higher than 0.5µg/L, which indicates the decline of the blood brain barrier.^13^ MMP-9 levels have a positive correlation with the severity of brain tissue inflammation.^14^ The concentration of NSE in ectocinerea neurons is approximately 2-4 times that in peripheral neurons. Increased serum levels of NSE indicates injury to the central nervous system.^15^ For patients undergoing cardiac surgery, brain injury may cause the decline of the blood brain barrier and the release of NSE and MMP-9 into the blood.^16^

In this study, the POCD incidence of Group B at 12 h and 24 h after the operation was significantly lower than that of Group A (*p*<0.05). Thus, the use of propofol for anesthesia maintenance in patients undergoing cardiac operation with PCB could prevent POCD and improve long-term prognosis. Propofol can effectively inhibit neuronal NMDA receptors, restrain internal flow of Ca2+ and avoid intracellular calcium overload, relieving the aerobic metabolism rate of the central nervous system.^17^ In addition, propofol can inhibit neuronal apoptosis and protect cognitive function by enhancing the activity of the mitochondrial mitoK-ATP channel, regulating Bax and Bcl-2 protein levels and inhibiting the expression of caspase-3.^18^

Furthermore, we found that the incidence of low cardiac output and thoracotomy bleeding had no significant differences between Group A and B. Thus, the application of propofol did not aggravate postoperative adverse events compared with sevoflurane. The results of our study are consistent with the previous report.^12^

## CONCLUSION

In summary, compared with sevoflurane, the use of propofol for anesthesia maintenance can effectively improve postoperative cognitive function of patients undergoing cardiac surgery with CPB and is also correlated t reduced serum levels of MMP-9, NSE and S100β.

### Authors’ Contributions

**ST, TX:** Designed this study and prepared this manuscript.

**FY, KZ:** Performed this study.

**WC:** Collected and analyzed clinical data.
